# Characterizing the Relationship Between Neutralization Sensitivity and *env* Gene Diversity During ART Suppression

**DOI:** 10.3389/fimmu.2021.710327

**Published:** 2021-09-15

**Authors:** Andrew Wilson, Leyn Shakhtour, Adam Ward, Yanqin Ren, Melina Recarey, Eva Stevenson, Maria Korom, Colin Kovacs, Erika Benko, R. Brad Jones, Rebecca M. Lynch

**Affiliations:** ^1^Lynch Lab, Department of Microbiology, Immunology, and Tropical Medicine, The George Washington University School of Medicine and Health Sciences, Washington, DC, United States; ^2^Jones Lab, Department of Medicine, Division of Infectious Diseases, Weill Cornell Medicine, New York, NY, United States; ^3^PhD Program in Epidemiology, The George Washington University Milken Institute School of Public Health, Washington, DC, United States; ^4^Department of Internal Medicine, Maple Leaf Medical Clinic, Toronto, ON, Canada

**Keywords:** broadly neutralizing antibodies, HIV-1, diversity, *env* gene, autologous antibodies, ART suppression

## Abstract

Although antiretroviral therapy (ART) successfully suppresses HIV-1 replication, ART-treated individuals must maintain therapy to avoid rebound from an integrated viral reservoir. Strategies to limit or clear this reservoir are urgently needed. Individuals infected for longer periods prior to ART appear to harbor more genetically diverse virus, but the roles of duration of infection and viral diversity in the humoral immune response remain to be studied. We aim to clarify a role, if any, for autologous and heterologous antibodies in multi-pronged approaches to clearing infection. To that end, we have characterized the breadths and potencies of antibody responses in individuals with varying durations of infection and HIV-1 envelope (*env)* gene diversity as well as the sensitivity of their inducible virus reservoir to broadly neutralizing antibodies (bNAbs). Plasma was collected from 8 well-characterized HIV-1^+^ males on ART with varied durations of active infection. HIV *envs* from reservoir-derived outgrowth viruses were amplified and single genome sequenced in order to measure genetic diversity in each participant. IgG from plasma was analyzed for binding titers against gp41 and gp120 proteins, and for neutralizing titers against a global HIV-1 reference panel as well as autologous outgrowth viruses. The sensitivity to bNAbs of these same autologous viruses was measured. Overall, we observed that greater *env* diversity was associated with higher neutralizing titers against the global panel and also increased resistance to certain bNAbs. Despite the presence of robust anti-HIV-1 antibody titers, we did not observe potent neutralization against autologous viruses. In fact, 3 of 8 participants harbored viruses that were completely resistant to the highest tested concentration of autologous IgG. That this lack of neutralization was observed regardless of ART duration or viral diversity suggests that the inducible reservoir harbors ‘escaped’ viruses (that co-evolved with autologous antibody responses), rather than proviruses archived from earlier in infection. Finally, we observed that viruses resistant to autologous neutralization remained sensitive to bNAbs, especially CD4bs and MPER bNAbs. Overall, our data suggest that the inducible reservoir is relatively resistant to autologous antibodies and that individuals with limited virus variation in the *env* gene, such as those who start ART early in infection, are more likely to be sensitive to bNAb treatment.

## Introduction

Although effective antiretroviral therapy (ART) suppresses HIV-1 replication, ART-treated individuals must maintain life-long therapy to avoid rebound from a persistent viral reservoir, and may experience adverse effects. This long-lived virus reservoir of integrated provirus poses an obstacle to curing HIV-1, and a deeper qualitative understanding of its composition may hold clues for improving therapeutic as well as cure strategies. Antibodies mediate effector functions such as neutralization and opsonization that could aid in suppressing virus replication, clearing infected cells, and boosting immune responses (1). Anti-HIV-1 antibodies could therefore, be used to prevent mother to child transmission (2) or reformatted as bi-specific or tri-specific molecules (3). The characterization of broadly neutralizing antibodies (bNAbs) capable of recognizing genetically diverse HIV-1 Env proteins has led to robust exploration of how to effectively use antibodies against the HIV-1 reservoir. To date, the results of clinical trials passively infusing bNAbs as IgG1 into chronically infected participants have been modest (4–10). Infusion with a single bNAb can increase time to rebound during analytic treatment interruption (ATI) (4, 10) or reduce viral load in participants not on suppressive ART (5, 6, 9), and these effects are improved with combination therapy (7, 8). The modest nature of these effects may reflect the presence of virus strains that are either completely resistant to the infused bNAb or insufficient antibody concentration and/or penetration. Ultimately the data suggest that the virus becomes sufficiently resistant to replicate faster than the available concentration of bNAb can neutralize. One clear lesson from these trials is that bNAbs were more effective when participants were prescreened for neutralization sensitivity, clearly indicating that further methods for overcoming bNAb resistance are needed.

HIV-1 sexual transmission often begins with a single founder virus (11–13) that diversifies over the course of infection resulting in a diverse quasispecies (14, 15). This genetic diversity is reflected in the integrated proviral reservoir as reviewed in (16) and is a consequence of rapid virus mutation during replication as well as selection pressure exerted by the immune system. In particular, the autologous antibody response exerts pressure on the HIV-1 *env* gene (17–20). Therefore, there is a circular relationship after virus transmission that starts with an antibody response that drives viral diversification, and results in escape from the antibody response. This inherent tension between the host immune system and virus replication is frequently called an “arms race” (21), but the effects of the arms race on efficacy of bNAb treatment during chronic infection remain unknown. We therefore perceived a need to define the complicated relationship between genetic diversity of *env*, duration of active viral infection, autologous antibody titers and reservoir virus sensitivity to bNAbs. We hypothesized longer durations of HIV-1 replication before ART suppression would lead to higher autologous antibody titers and to greater Env diversity within the reservoir, which would be associated with increased resistance to bNAbs.

## Materials and Methods

### Study Participants

All participants were HIV-1 infected males on ART recruited from the Maple Leaf Medical Clinic in Toronto, Canada, through a protocol approved by the University of Toronto Institutional Review Board. Secondary use of the samples from Toronto was approved through the George Washington University Institutional Review Boards. All subjects were adults and gave written informed consent. Clinical data for these participants are described in **Table 1**. We calculated an estimated date of infection to be midway between the last negative test and first positive test. The estimated duration of active infection was calculated to be the months between the estimated date of infection and the date of documented ART initiation.

**Table 1:** Clinical characteristics of study cohort.

Participant ID	Age	Sex	Viral Load (copies/ml)	CD4 Count (cells/mm^3^)	IUPM	HIV Subtype	Estimated duration of unsuppressed infection (months)	Duration of ART (years)	QVOA wells (number)
OM5334	33	Male	Undetectable	812	1.57	B	3	3	5
OM5267	29	Male	Undetectable	429	2.34	B	10.5	3	4
OM5346	48	Male	Undetectable	1182	0.27	B, AG	25.5	5	4
OM5148	47	Male	Undetectable	733	1.02	B	69	10	5
CIRC0196	56	Male	Undetectable	679	0.49	B	81.5	3	5
OM5162	53	Male	Undetectable	478	0.65	B	>3	14	5
OM5001	43	Male	42	540	10.46	B	>14	9	4
OM5365	56	Male	Undetectable	624	0.42	B	>18	25	3

### QVOA

Leukapheresis was performed on ART-treated participants. Peripheral blood mononuclear cells (PBMCs) were isolated by centrifugation with Ficoll (GE Life Sciences). CD4 T cells were enriched from PBMCs (Stemcell Technologies). Cells were serially diluted (2 million, 1 million, 0.5 million, 0.2 million, and 0.1 million cells/well) and plated in 24-well plates, with 12 replicates at each concentration. Phytohemagglutinin (PHA) and irradiated allogenic HIV-negative PBMCs were added to activate the CD4 T cells. CCR5^+^ MOLT-4 cells [NIH AIDS reagent program (22)] were added 24 hours later for viral replication. Media were changed every 3-4 days and ELISA for p24 protein was performed after 14 days of culture as described in (23). In general, supernatants were collected from p24^+^ wells for which fewer than 50% of dilution replicates were positive. To increase the volume of outgrowth virus available for use, viruses were re-grown for 6 days by infecting and proliferating in new HIV-negative CD4^+^ T cells.

### Single Genome Sequencing

SGS was performed as previously described (24)} with the following modifications. Viral RNA was extracted from culture supernatants by QIAmp kit (Qiagen, Germantown MD). cDNA was synthesized as previously described in (9), and *env* genes were amplified by nested PCR using the Platinum Taq High Fidelity polymerase (Invitrogen). Template cDNA was serially diluted so that fewer than 33% of PCR replicates were positive, ensuring that a majority of amplicons would be generated from a single cDNA template. Well-described primers Env_outF1 (TAGAGCCCTGGAAGCATCCAGGAAG) and Env_outR1 (TTGCTACTTGTGATTGCTCCATGT) were used for first round amplification, and Env_inF2 (CACCTTAGGCATCTCCTATGGCAGGAAGAAG) and Env_inR2 (GTCTCGAGATACTGCTCCCACCC) for the second round. In the case of Subtype AG viruses from participant OM5346, customized primer Env_innR3 (GATACTGCTCCCACCCCATCTGC) was used in lieu of Env_inR2. All PCR mixes were generated in PCR clean rooms free of post-PCR or plasmid DNA. Amplicons were run on 1% agarose gels and sequenced by ACGT Inc. A minimum of three single-template sequences were obtained from each well. Sequences that contained stop codons, large deletions, or mixed bases were removed from further analysis.

### Maximum Likelihood Trees

All QC’ed sequences were translation aligned by participant to generate individual nucleic acid alignments. Due to the additive chances of mutation during replication in the QVOA assay, cDNA synthesis, and PCR (25–27), wells were considered to contain a single virus if no sequence contained more than four amino acid mutations from the consensus sequence for that well, and that each mutation was unique to that sequence. These consensus sequences for all single-virus wells were translated and protein sequences from all participants were aligned with MUSCLE to Consensus B and AG sequences (www.hiv.lanl.gov). All alignments were hand-edited and gap-stripped for regions that could not unambiguously be aligned. All sequence analysis was performed in the Geneious suite version (9.0.5) [http://www.geneious.com (28)]. Maximum likelihood trees were generated from these alignments using RAxML-HPC BlackBox (8.2.12) run on the Cyberinfrastructure for Phylogenetic Research (CIPRES) Science Gateway. Trees are rooted on midpoint for visualization using MEGA version X (29, 30). Bootstraps greater than 90 are shown.

### Average Pairwise Distance

Average pairwise distance was calculated using DIVEIN (https://indra.mullins.microbiol.washington.edu/DIVEIN/diver.html). Briefly, consensus protein sequences for viruses from each individual were aligned with MUSCLE. Alignments were input into DIVEIN using the HIVb substitution model to generate average pairwise distance for each individual.

### ELISA

IgG purification from plasma was performed with the Melon Gel IgG purification kit (Thermo Fisher) according to the manufacturer’s instructions. ELISAs against recombinant proteins YU2 gp120 (provided by Dr. John Mascola) and DIII gp41 (Abcam) were run as previously described (31), with the following modifications. IgG was diluted to 50μg/mL and serially diluted 5-fold to 0.0032μg/mL in B3T buffer (150 mM NaCl, 50 mM Tris-HCl, 1 mM EDTA, 3.3% fetal bovine serum, 2% bovine albumin, 0.07% Tween 20). Briefly, 96-well ELISA plates were coated with 2 μg/ml of the specified recombinant protein in phosphate-buffered saline (PBS) overnight at 4°C. The following day, the plates were blocked with B3T buffer. IgGs were detected using peroxidase-conjugated goat anti-human IgG antibody (Jackson ImmunoResearch). All incubations were for 1 h at 37°C, and all volumes were 100 μl, except for the blocking step, which was 200 μl. The plates were washed between incubations with 0.1% Tween 20 in PBS, detected using SureBlue TMB substrate (SeraCare, VWR), and subsequently read at 450 nm.

### Neutralization Assay

Single-round neutralization assays in Tzm-bl target cells [NIH AIDS Reagent Program (32)] were performed as described previously (23, 31, 33). Briefly, neutralization activity of isolated plasma IgG samples were tested against both heterologous HIV-Env pseudotyped viruses and replication-competent autologous outgrowth viruses. IgG was diluted to 50μg/mL and serially diluted 5-fold to 0.0032μg/mL in duplicate. Plasmids for pseudovirus production were from the global panel of 12 reference *env* clones [NIH AIDS Reagent Program (34)]. Outgrowth viruses were kept in the presence of 3.5uM Indinavir (Sigma-Aldrich) to prevent replication. Viruses were titered on Tzm-bl target cells before use and diluted to target 5500 relative luciferase units (RLU) for virus isolates (which were very low volume) and 45,000 RLU for pseudovirus. Virus and antibody pairings were incubated at 37°C for 30 min in 96-well clear flat-bottom black culture plates (Greiner Bio-One) before Tzm-bl cells were added at a concentration of 10^4^ per well in the presence of DEAE-Dextran (Sigma Aldrich) diluted to 20μg/ml. After 48 hours, plates were read by removing 100μl of media from each well, and adding 100 μl of SpectraMax Glo Steady-Luc reporter assay reagent (Molecular Devices). Luminescence intensity was measured using a SpectraMax i3x multimode detection platform (Molecular Devices). per the manufacturer’s instructions. Neutralization curves were generated after background subtraction of mean RLU of cell only wells by calculating the change in RLUs in the presence of antibody to the mean RLU of virus-only control wells. Curves were fit by nonlinear regression using the asymmetric five-parameter logistic equation in Prism 9 (GraphPad). The 50% and 80% inhibitory concentrations (IC_50_ and IC_80_) were defined as the antibody dilutions that neutralize 50% and 80% of the virus, respectively.

### Statistics

All statistical analyses were performed in Prism 9 (GraphPad). Comparisons were performed using Mann Whitney and correlations using non-parametric Spearman.

## Results

### Association Between Genetic Diversity and bNAb Sensitivity

**Figure 1:**
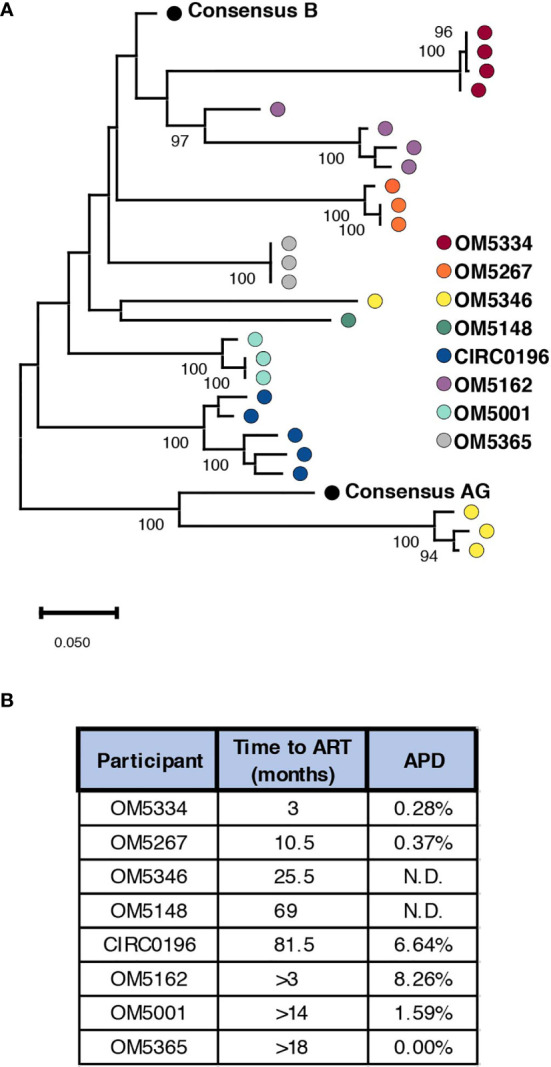
Genetic diversity of participant reservoir virus increases with active infection length. **(A)** A minimum of 3 SGS sequences were used to generate consensus sequences for each QVOA well. The protein sequence of each well was aligned to consensus subtype B and AG and a maximum likelihood tree was generated. Wells containing multiple viruses were excluded. Each dot represents one QVOA outgrowth virus well and is color-coded by participant. The tree is midpoint rooted for visualization and bootstraps over 90% are shown. **(B)** For all participants a minimum duration of infection was calculated using the date of diagnosis and date of ART initiation. For individuals for whom an HIV^-^ date was confirmed, an estimated length of infection was calculated using an estimated date of infection as the midpoint between confirmed HIV^-^ and HIV^+^ time points. For each person, these estimated times of active infection are listed next to the average pairwise distance (APD) of Env for their inducible reservoir viruses.

To successfully contribute to curing HIV-1, bNAbs will need to both neutralize circulating virus to prevent new infections, while also tagging infected cells displaying viral antigen for killing by immune cells. Previously we studied the abilities of bNAbs to recognize both cell-free HIV virions as well as matched infected cells from the inducible reservoir of 8 individuals. From these studies, we observed both inter-and intra-individual variation in bNAb sensitivity (23). Earlier studies have indicated that diversity within the persistent HIV reservoir increases with duration of active infection (13, 15, 35, 36), but it remains unknown how this diversity could affect sensitivity to bNAb neutralization. To study this question, we performed single genome sequencing (SGS) on the 35 quantitative virus outgrowth assay (QVOA) cultures from these 8 individuals (**Table 1**). These 8 males represented a wide range of reservoir sizes as measured by QVOA in infectious units per million cells (IUPM) (**Table 1**). A minimum of 3 *env* sequences were generated from each of the 3-5 QVOA supernatants. Outgrowth wells were considered to contain a single virus if all amplified sequences were within 4 amino acids of the consensus and all mutations from consensus were unique (i.e., not fixed in multiple sequences). We chose this definition to allow for mutations that may have been caused during viral outgrowth, reverse transcription, or PCR amplification (25–27). The majority (77%) of QVOA wells contained only a single virus by this criterion, and wells containing multiple viruses were excluded from further analyses (**Supplementary Figure 1**). We performed these analyses to ensure we only studied wells containing a single virus, so that we could pair our functional data to our genetic data and be confident that the sequence studied was indeed the one being tested *in vitro*. In particular, QVOA outgrowths for OM5148 contained multiple viruses in 4 out of the 5 wells, leading to only one well that passed our criteria for further phenotypic and phylogenetic studies. For all wells containing a single virus, consensus sequences were generated and aligned to produce a maximum-likelihood tree of all Env proteins (**Figure 1A**). This sequencing revealed that OM5346 was co-infected with both subtype B and AG viruses, introducing a secondary source of diversity beyond viral evolution.

We next quantified genetic diversity for each individual by calculating average pairwise distance (APD) between the consensus protein sequences of their QVOA wells. OM5148 and OM5346 were excluded from this analysis for either having only one sequence or multiple subtypes respectively; therefore, we could only calculate APD for 6 of 8 participants (**Figure 1B**). APDs varied among individuals, ranging from 0% to 8.26%, but appeared to group into “high” diversity with APDs greater than 6% (CIRC0196 and OM5162) or “low” diversity with APDs less than 2% (OM5334, OM5267, OM5001 and OM5365). We hypothesized that diversity was linked to duration of actively replicating virus before ART suppression. For 5 individuals there were clinical records documenting an HIV-1 negative test date. We calculated an estimated date of infection to be midway between the last negative test and first positive test. We then estimated the duration of active infection to be the months between the estimated date of infection and the date of documented ART initiation. Of these 5 individuals, only 3 had calculated APDs because of lack of sequences or multiple subtypes in the other individuals. The 2 individuals with an estimated length of infection of less than a year were in the low diversity group, and the person with an estimated length of greater than six years was in the high diversity group (**Figure 1B**). These observations are consistent with previous literature demonstrating that length of infection correlates with increased genetic diversity (13, 15, 35, 36).

**Figure 2:**
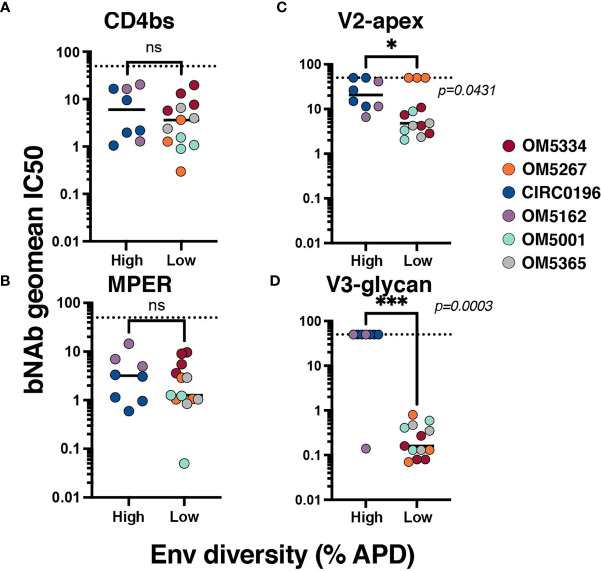
Association between reservoir diversity and bNAb resistance. Each virus was tested for neutralization sensitivity against a panel of 10 HIV-1 bNAbs. The geometric IC_50_ of each individuals’ virus-bNAb pairing was calculated by bNAb epitope: **(A)** CD4bs antibodies include VRC01, VRC07-523, 3BNC117, and N6, **(B)** MPER antibody 10E8v4-V5F-100cF (because there is only one bNAb, IC_50_ is plotted), **(C)** V2-apex antibodies PGDM1400, CAP256.VRC26.25, and PG09, and **(D)** V3-glycan targeting antibodies PGT121 and 10-1074. Geometric mean IC_50_s for each virus are color-coded by individual and grouped by high (>6%APD) or low (<2% APD) Env diversity. Comparisons were performed by Mann-Whitney. * signifies p < 0.05. *** signifies p < 0.001. ns signifies p > 0.05.

To investigate the relationship between genetic diversity and bNAb sensitivity, we re-analyzed data on the neutralization sensitivity of these QVOA outgrowths to 10 bNAbs (23). We could now exclude wells that contained multiple viruses because of the sequencing analyses. To quantify the bNAb sensitivity of each individuals’ reservoir, the geometric mean IC_50_ for all outgrowth viruses against bNAbs was calculated (**Supplementary Table 1**). When graphed by APD, the geometric mean IC_50_s of high diversity individuals’ viruses were not significantly different from the low group when tested against CD4bs bNAbs (**Figure 2A**) or 10E8v4-V5F-100cF (**Figure 2B**). There are significant differences between these groups, however, for bNAbs targeting V2-apex (**Figure 2C**) and V3-glycan (**Figure 2D**) epitopes. High diversity people exhibited more resistance to these bNAbs, although it is important to note that OM5267 has very low diversity and is completely resistant to the anti-V2-apex bNAbs.

### Association Between Genetic Diversity and Autologous Antibody

**Figure 3:**
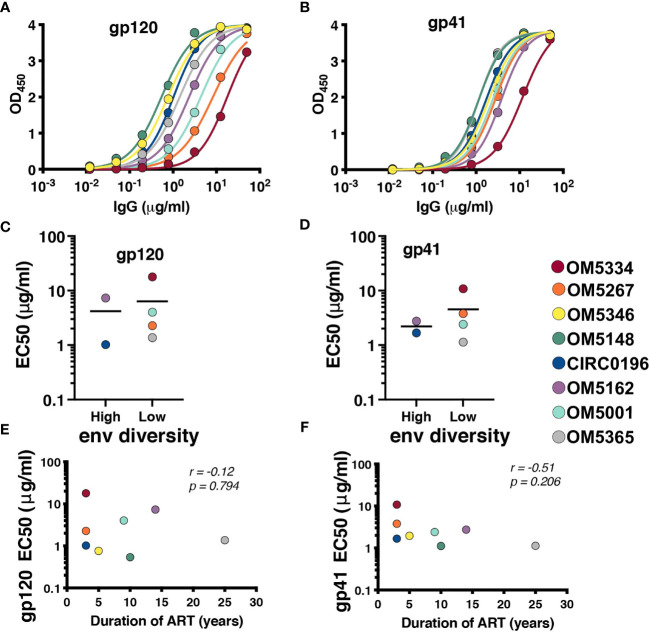
All participants exhibit detectable anti-HIV antibodies. IgG binding for each person was assessed by indirect ELISA against **(A)** YU2 gp120 and **(B)** DIII gp41. EC_50_s for each participant against gp120 **(C)** and gp41 **(D)** were graphed by high (>6%APD) and low (<2% APD) Env diversity. EC_50_s for gp120 **(E)** and gp41 **(F)** were graphed against duration of ART. Comparisons were performed by Mann-Whitney and correlations by nonparametric Spearman.

Recent papers have highlighted the role of autologous antibodies in restricting virus from rebounding *in vivo* after ATI (37, 38). To study the titers of autologous antibodies in each individual, we first isolated IgG from plasma because circulating ART can confound anti-viral antibody assays. We next measured binding titers against subtype B HIV-1 gp120 (**Figure 3A**) and gp41 proteins (**Figure 3B**). All participants exhibited similar gp41 binding curves with slightly more variation in binding to gp120. Titers were quantified by calculating EC_50_ values, and there was no apparent difference between people with high or low *env* diversity (**Figures 3C, D**). There was also no correlation between gp120 (**Figure 3E**) or gp41 (**Figure 3F**) titers and duration of ART in our study samples.

**Figure 4:**
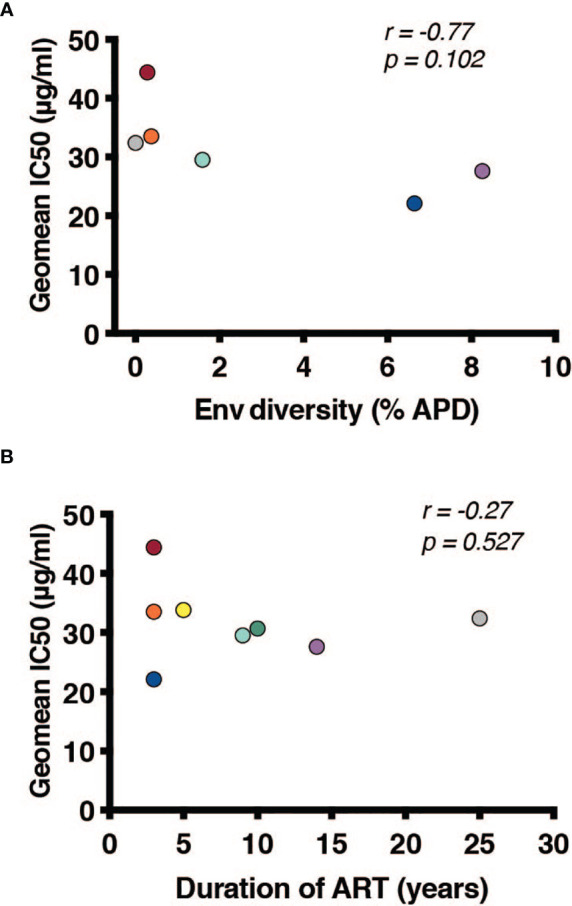
Duration of infection and reservoir diversity are associated with neutralization of global HIV-1 viruses. Geometric mean IC_50_ of IgGs against a global HIV-1 reference panel were calculated for each individual and graphed against **(A)** Env diversity and **(B)** duration of ART. Correlations were calculated by nonparametric Spearman.

Once it was established that all individuals had robust antibody titers despite years of ART suppression (ranging between 3 to 25), we next tested their abilities to neutralize virus on a well-established global reference panel of HIV-1 pseudoviruses (34). On average, our study participants neutralized 65.6% of these viruses, but this neutralization was typically not potent, with a geometric mean IC_50_ of 25.5μg/mL for neutralized viruses (**Table 2**). These neutralization patterns are consistent with antibody responses during chronic infection (34, 39, 40). We assigned an overall titer on the global panel for each person by calculating a geometric mean IC_50_ for all viruses, designating resistant viruses “50”. We observed an apparent trend toward increased breadth and potency (lower geometric mean IC_50_) associated with increased *env* diversity (r = -0.77; **Figure 4A**), but no detectable trend between this overall IC_50_ and duration of ART (r = -0.27; **Figure 4B**).

**Table 2:** IC_50_s of IgG on a 12-virus panel.

IC50 (μg/ml)	*Subtype B*		*Subtype C*		*Subtype A*			*Subtype BC*			*Subtype AE*		*Subtype G*				
*Tier*	*2*	*2*	*1B or 2*	*2*	*2*	*2*		*2*	*2*	*2*	*2 or 3*	*2*	*2*				
Ab Sample	TRO11	X2278	25710	CEO217	CE1176	398F1		246F3	BJOX2000	CH119	CNE8	CNE55	X1632	SIV_mac239_	GeoMean of Neutralized Virus	Percent of viruses neutralized	GeoMean of all viruses
**OM5334**	42.1	40.7	>50	47.4	>50	18.4		49.7	>50	>50	>50	>50	>50	>50	37.5	41.7	44.4
**OM5267**	35.9	44.5	45.3	46.6	19.5	15.3		45.1	>50	>50	>50	22.5	15.6	>50	29.3	75.0	33.5
**OM5346**	23.0	45.6	>50	14.8	38.8	15.0		>50	43.7	>50	>50	>50	18.4	>50	25.6	58.3	33.8
**OM5148**	8.11	>50	>50	49.7	38.7	15.3		49.5	>50	>50	>50	16.6	11.5	>50	21.7	58.3	30.7
**CIRC0196**	21.7	8.78	>50	>50	30.9	8.33		10.8	18.9	>50	18.8	18.1	32.7	>50	16.9	75.0	22.1
**OM5162**	23.5	9.65	>50	46.7	19.1	17.7		36.9	20.5	>50	45.5	35.8	17.6	>50	24.5	83.3	27.6
**OM5001**	>50	10.5	14.8	46.9	36.8	17.1		10.8	>50	>50	46.3	45.7	33.3	>50	24.8	75.0	29.5
**OM5365**	10.2	46.9	>50	12.0	>50	15.5		>50	>50	>50	48.8	21.0	46.1	>50	23.7	58.3	32.4

IC (µg/ml) >50 10-50 1-10 0.1-1 <0.1

**Figure 5:**
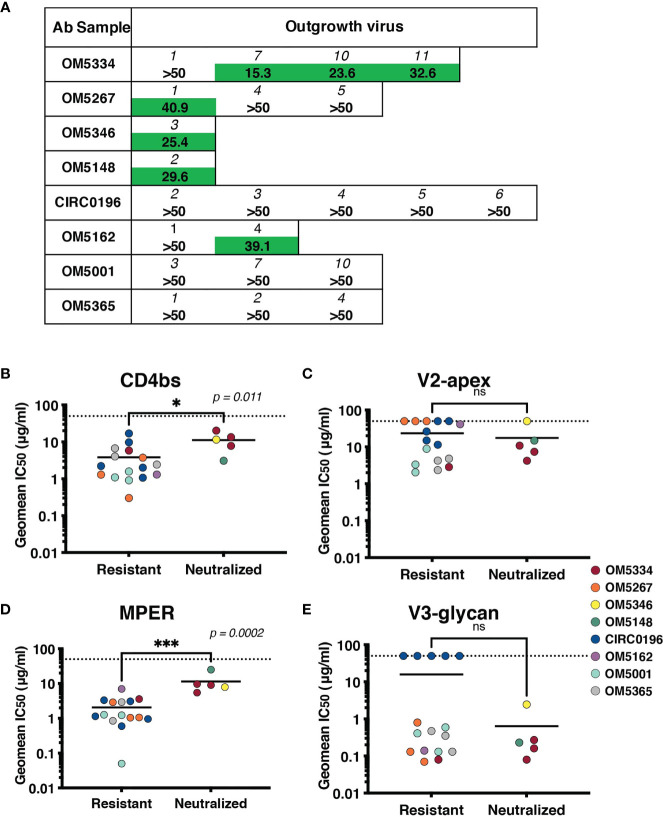
Sensitivity of the inducible reservoir to autologous IgG and to bNAbs targeting multiple epitopes. **(A)** IC_50_ values for each individual’s IgG against autologous QVOA-derived viruses. Viruses resistant at 50µg/mL of IgG were denoted with “>50”. **(B–E)** Geometric mean IC_50_s of bNAbs were calculated for viruses resistant to autologous neutralization (IC_50_ >35µg/mL) and those that were sensitive. IC_50_s were graphed by epitope targeted: **(B)** CD4bs antibodies include VRC01, VRC07-523, 3BNC117, and N6, **(C)** V2-apex antibodies include PGDM1400, CAP256.VRC26.25, and PG09, **(D)** MPER antibody 10E8v4-V5F-100cF, and **(E)** V3-glycan antibodies include PGT121 and 10-1074. Geometric mean IC_50_s are color-coded by individual, and the limit of detection (50μg/ml) is indicated with a dotted line. * signifies p < 0.05. *** signifies p < 0.001. ns signifies p > 0.05.

To assess if these IgGs were effective against autologous viruses, we performed neutralization assays against the same QVOA outgrowth cultures we had sequenced. Importantly, these polyclonal IgGs were derived from the plasma samples matched to the leukapheresis cells used in the QVOA. Therefore, these were the antibodies circulating at the time point from which the reservoir viruses were isolated. Interestingly, across 22 pairings of virus and autologous IgG, we observed no instances of potent neutralization, suggesting that these viruses were mostly escaped from circulating antibodies (**Figure 5A**).

### Combination of Autologous and Heterologous Antibody Sensitivity

Recent studies have demonstrated that autologous neutralization can restrict which viruses rebound after ATI (37, 38). Therefore, viruses that have not completely escaped the autologous antibody response can be neutralized by these antibodies, and bNAb therapy would need to particularly target autologous-resistant virus. One of these studies demonstrated that autologous neutralization with an IC_50_ less than 35 μg/ml was sufficient to block outgrowth in the QVOA (38). We therefore, re-analyzed our data using this suggested cutoff for autologous resistance. We measured sensitivity to bNAbs targeting the CD4bs (**Figure 5B**), V2-apex (**Figure 5C**), MPER (**Figure 5D**) or V3-glycan (**Figure 5E**) by both autologous-resistant or sensitive viruses. Interestingly although there was no difference in sensitivity to bNAbs targeting the variable loop epitopes such as V3-glycan or V2-apex, viruses resistant to autologous neutralization were more sensitive to CD4bs antibodies (p = 0.011) and MPER-targeting antibody10E8-v4-V5R-100cF (p = 0.0002).

## Discussion

Genetic diversity in the HIV-1 reservoir poses challenges for long-term supplemental therapies to current ART and cure strategies. To gain insight into how this diversity might be overcome to improve antibody efficacy, we first explored the role of ART suppression in decreasing the overall diversity of the viral quasispecies. We found that 2 individuals with an estimated duration of active viral replication before ART suppression of less than a year had ‘low’ diversity whereas an individual with an estimated duration of infection of greater than six years had ‘high’ diversity. Our limited sample size precluded correlation analysis but is consistent with earlier studies (13, 15, 35, 36). We extended these findings to assess how Env diversity related to bNAb neutralization. Overall, geometric mean IC_50_s to CD4bs or MPER bNAbs were not significantly different between individuals with high or low Env diversity, but those with high diversity were more likely to be resistant to V2-apex and V3-glycan bNAbs. Interestingly, these are two epitopes highly affected by glycosylation, and in fact glycans comprise part of the epitope for some of these antibodies. Therefore, it seems plausible that longer duration of infection might affect glycans associated with bNAb neutralization (41, 42). These data suggest that individuals with shorter active infections, and by extension less reservoir diversity, may be more effectively treated with bNAbs. However, OM5267 did not follow this trend, and was completely resistant to V2 antibodies despite very low Env diversity. This observation highlights that even people who started ART early may harbor bNAb resistant viruses, but it may be easier to pre-screen for this resistance before enrolling in a clinical trial as there is less likelihood of a minor resistant variant existing.

The role of autologous antibody responses is another area of interest when considering cure strategies. During the course of infection, antibodies generated against HIV-1 are insufficient to prevent the spread of the virus, but can typically neutralize viruses from earlier timepoints in the infection (17, 18, 42, 43). Similarly, these antibodies are inadequate to prevent rebound during an interruption of ART suppression; however, autologous antibodies likely play a selective role in restricting which viruses rebound from latently infected cells (37, 38). It also remains incompletely understood how stable antibody responses remain during ART. It is known that individuals who are suppressed early after HIV-1 infection have low antibody titers (44), and it has been observed that titers wane over time during durable ART suppression, but not dramatically (45). We therefore hypothesized that longer ART suppression would correlate with lower antibody titers. In testing isolated IgG for the ability to bind and neutralize heterologous virus, we observed detectable binding titers and ‘average’ neutralization, suggesting that autologous antibodies are both present and functional irrespective of length of ART treatment – even up to 25 years. It is important to note that our samples are cross-sectional, and without longitudinal time points, we cannot directly address this question of durability. We did observe a trend for individuals with greater reservoir diversity and longer durations of infection to have increased breadth and potency against a global HIV-1 reference panel. This trend was not statistically significant, likely because of our small sample size, but is consistent with studies of breadth development during chronic infection (46–49).

After establishing that HIV-1 specific antibodies were still detectable and circulating, we examined their efficacy against the participants’ own inducible reservoir viruses. We observed no instances of potent neutralization, and indeed, many viruses were completely resistant to autologous IgG at the highest concentration tested (50 µg/mL). It is important to note that these viruses were not cloned, and are T-cell derived, which can result in increased resistance to antibody neutralization, likely due to the number of HIV trimers expressed on the virion as well as glycosylation patterns (50–53). Our data are consistent with other reported autologous neutralizing antibody titers from chronically suppressed individuals (38) and suggest that the viruses have mainly evolved away from the circulating antibodies and are already escaped or nearly-escaped. These data are consistent with descriptions of autologous neutralization during active chronic infection (17, 18, 42, 43), and therefore, also consistent with reports that the majority of the reservoir is established at the time of ART initiation (54). If the inducible reservoir did harbor archival variants from earlier infection timepoints, we would expect to see more potent autologous neutralization. Because we were not able to deeply sample the reservoir, we cannot exclude the possibility that rare, sensitive variants also exist. Finally, we investigated the potential cooperation between autologous antibodies and bNAbs by measuring bNAb sensitivity of autologous-resistant viruses. If autologous antibodies can restrict some proportion of viruses from rebounding from the integrated reservoir, then potentially bNAb therapy could focus on autologous-resistant viruses. Because of our small sample size, we observed only 5 outgrowth viruses that were neutralized by autologous antibodies, 3 of which were from a single individual. Nevertheless, these viruses were more resistant to CD4bs antibodies and MPER-targeting antibody 10E8v4-V5F-100cF. It would be of interest to examine if CD4bs and MPER bNAbs would in fact better restrict autologous-resistant viruses.

In conclusion, our study finds that antibody titers and functionality during ART suppression are quite similar to those described during chronic infection. We found that the inducible reservoir from these 8 individuals studied was fairly refractory to autologous neutralization. This observation is consistent with the reservoir being comprised of viruses that had co-evolved with these antibodies and therefore, were mostly escaped. We also observed a trend toward individuals with shorter active infections, and by extension less reservoir diversity, harboring reservoir viruses more sensitive to bNAbs. These data suggest that individuals able to suppress virus replication with ART early in infection may be more effectively treated with bNAbs, and that particular care should be taken when screening for bNAb resistant variants in individuals who were infected for longer periods of time before starting ART.

## Data Availability Statement

All sequence data is submitted to GenBank with accession numbers OK011845-OK011988.

## Author Contributions

MK, CK, EB, and YR contributed data. AWa performed data analysis. AWi, LS, MR, and ES collected data. RJ contributed data and helped write the paper. AWi and RL conceived and designed the experiments, performed analyses and wrote the paper. All authors contributed to the article and approved the submitted version.

## Funding

Research reported in this publication was supported by the National Institute of Allergy and Infectious Diseases of the National Institutes of Health under award number UM1AI126617 (to the Martin Delaney BELIEVE Collaboratory). It was also supported by a supplement from the District of Columbia Center for AIDS Research, an NIH funded program (P30AI117970), which is supported by the following NIH Co-Funding and Participating Institutes and Centers: NIAID, NCI, NICHD, NHLBI, NIDA, NIMH, NIA, NIDDK, NIMHD, NIDCR, NINR, FIC and OAR.

## Author Disclaimer

The content is solely the responsibility of the authors and does not necessarily represent the official views of the NIH.

## Conflict of Interest

The authors declare that the research was conducted in the absence of any commercial or financial relationships that could be construed as a potential conflict of interest.

## Publisher’s Note

All claims expressed in this article are solely those of the authors and do not necessarily represent those of their affiliated organizations, or those of the publisher, the editors and the reviewers. Any product that may be evaluated in this article, or claim that may be made by its manufacturer, is not guaranteed or endorsed by the publisher.

## Supplementary Material

Supplementary Figure 1 Single genome amplification sequences obtained from each individual. Maximum likelihood trees of all SGS sequences obtained for each individual. **(A)** Trees of individuals with low diversity (<2% predicted APD). Scale bars are 0.001 for these individuals, with the exception of OM5365 (grey), where the scale bar is 0.0002. **(B)** Trees for individuals with high diversity (>2% predicted APD). Scale bars are 0.01 for these individuals.
