# Supplementary figures and images for: Characterizing the Relationship Between Neutralization Sensitivity and env Gene Diversity During ART Suppression
Source: Front Immunol. 2021 Sep 15;12:710327. doi: 10.3389/fimmu.2021.710327 (PMC8479156; doi:10.3389/fimmu.2021.710327)

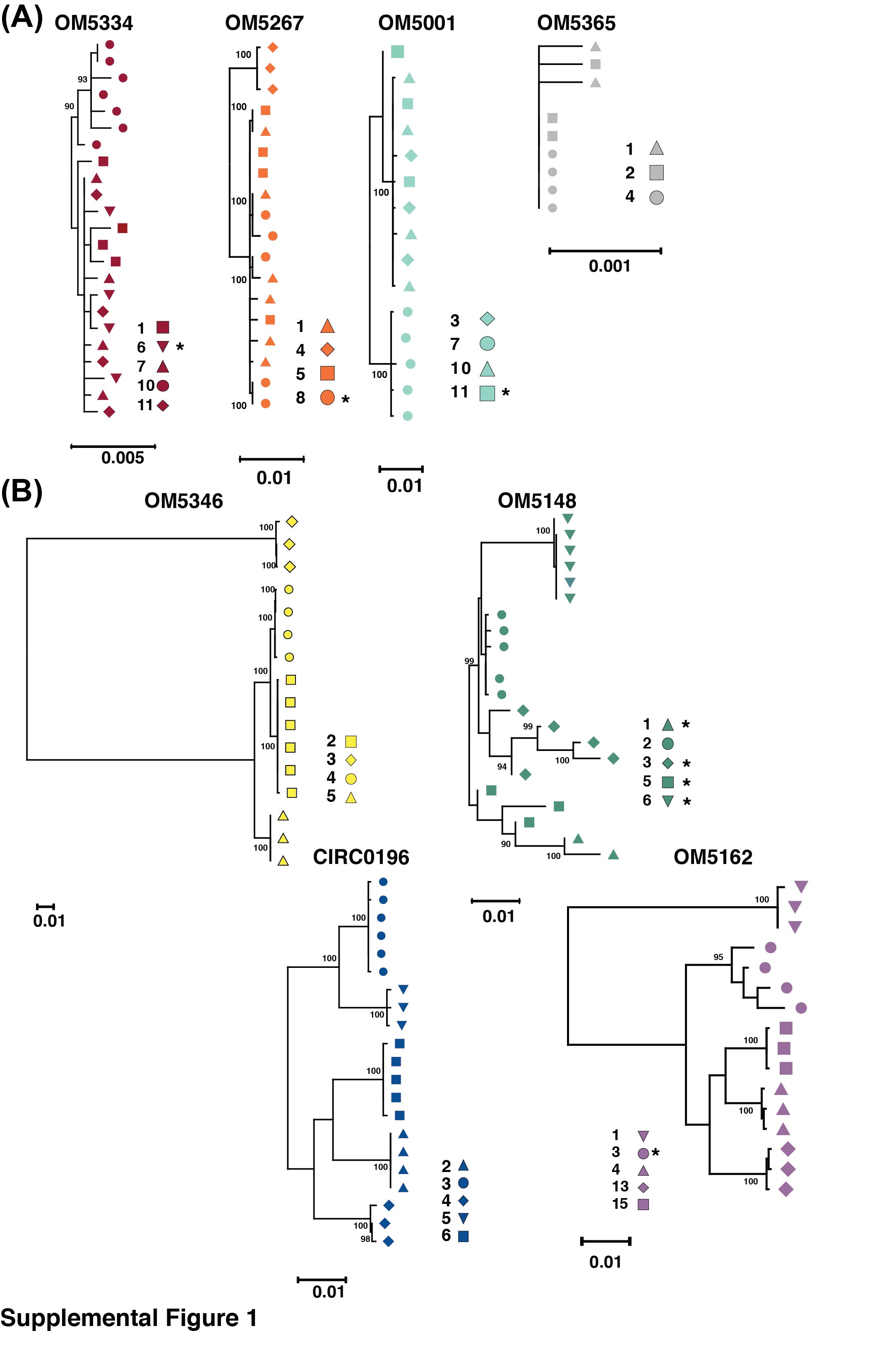

Supplement: Supplementary Figure 1 — Single genome amplification sequences obtained from each individual. Maximum likelihood trees of all SGS sequences obtained for each individual. (A) Trees of individuals with low diversity (<2% predicted APD). Scale bars are 0.001 for these individuals, with the exception of OM5365 (grey), where the scale bar is 0.0002. (B) Trees for individuals with high diversity (>2% predicted APD). Scale bars are 0.01 for these individuals. [file Image_1.jpeg]
